# Epileptogenic Zone Localization With ^18^FDG PET Using a New Dynamic Parametric Analysis

**DOI:** 10.3389/fneur.2019.00380

**Published:** 2019-04-17

**Authors:** Maria Mayoral, Aida Niñerola-Baizán, Berta Marti-Fuster, Antonio Donaire, Andrés Perissinotti, Jordi Rumià, Núria Bargalló, Roser Sala-Llonch, Javier Pavia, Domènec Ros, Mar Carreño, Francesca Pons, Xavier Setoain

**Affiliations:** ^1^Nuclear Medicine Department, Hospital Clínic, Barcelona, Spain; ^2^Biomedical Imaging Group, Biomedical Research Networking Center in Bioengineering, Biomaterials and Nanomedicine (CIBER-BBN), Barcelona, Spain; ^3^Biophysics and Bioengineering Unit, Biomedicine Department, School of Medicine, University of Barcelona, Barcelona, Spain; ^4^Neurology Department, Hospital Clínic, Barcelona, Spain; ^5^August Pi i Sunyer Biomedical Research Institute (IDIBAPS), Barcelona, Spain; ^6^Neurosurgery Department, Hospital Clínic, Barcelona, Spain; ^7^Radiology Department, Hospital Clínic, Barcelona, Spain

**Keywords:** epilepsy, functional neuroimaging, PET, SPM, parametric analysis, dynamic analysis

## Abstract

**Introduction:** [^18^F]fluorodeoxyglucose (^18^F-FDG) positron emission tomography (PET) is part of the regular preoperative work-up in medically refractory epilepsy. As a complement to visual evaluation of PET, statistical parametric maps can help in the detection of the epileptogenic zone (EZ). However, software packages currently available are time-consuming and little intuitive for physicians. We develop a user-friendly software (referred as PET-analysis) for EZ localization in PET studies that allows dynamic real-time statistical parametric analysis. To evaluate its performance, the outcome of PET-analysis was compared with the results obtained by visual assessment and Statistical Parametric Mapping (SPM).

**Methods:** Thirty patients with medically refractory epilepsy who underwent presurgical ^18^F-FDG PET with good post-operative outcomes were included. The ^18^F-FDG PET studies were evaluated by visual assessment, with SPM8 and PET-analysis. In SPM, parametric T-maps were thresholded at corrected *p* < 0.05 and cluster size k = 50 and at uncorrected *p* < 0.001 and k = 100 (the most used parameters in the literature). Since PET-analysis rapidly processes different threshold combinations, T-maps were thresholded with multiple *p*-value and different clusters sizes. The presurgical EZ identified by visual assessment, SPM and PET-analysis was compared to the confirmed EZ according to post-surgical follow-up.

**Results:** PET-analysis obtained 66.7% (20/30) of correctly localizing studies, comparable to the 70.0% (21/30) achieved by visual assessment and significantly higher (*p* < 0.05) than that obtained with the SPM threshold *p* < 0.001/k = 100, of 36.7% (11/30). Only one study was positive, albeit non-localizing, with the SPM threshold corrected *p* < 0.05/k = 50. Concordance was substantial for PET-analysis (κ = 0.643) and visual interpretation (κ = 0.622), being fair for SPM (κ = 0.242).

**Conclusion:** Compared to SPM with the fixed standard parameters, PET-analysis may be superior in EZ localization with its easy and rapid processing of different threshold combinations. The results of this initial proof-of-concept study validate the clinical use of PET-analysis as a robust objective complementary tool to visual assessment for EZ localization.

## Introduction

Surgical resection is the potentially curative treatment option in one third of epileptic patients who remain uncontrolled despite polytherapy with antiepileptic drugs. Interictal [^18^F]fluorodeoxyglucose (^18^F-FDG) positron emission tomography (PET) has traditionally been regarded as a complementary imaging modality in epilepsy, although some studies have reported similar post-operative outcomes based on PET in comparison with magnetic resonance imaging (MRI) (1–4). However, the sensitivity of PET on visual assessment to detect the epileptogenic zone (EZ) is moderate, being around 80% in temporal lobe epilepsy (TLE) and 60–70% in extratemporal lobe epilepsy (5–7). Visual interpretation of brain PET imaging can be improved by means of objective complementary software tools which compare each study to a normal control group using statistical parametric analysis. This is essential in epilepsy in which subtle hypometabolic changes may be overlooked by the naked eye. Software packages currently available for interpretation of brain imaging studies such as Statistical Parametric Mapping (SPM) (8) are of generic purpose and do not allow making real-time dynamic changes in the level of detectability. Furthermore, faster image processing and more intuitive software tools for physicians not familiar with programming language are required. Taking all of the above into account, the aim of this study was to develop, describe and validate a dedicated user-friendly software (referred in this article as PET-analysis) for EZ localization in ^18^F-FDG PET studies in patients with medically refractory epilepsy. To evaluate its performance, the outcome of PET-analysis was compared with the results obtained by visual assessment and SPM processing using the standard parameters from literature.

## Materials and Methods

### Subject Selection and Characteristics

Clinical records of adult patients with medically refractory epilepsy visited at our center for preoperative assessment were reviewed. Candidacy for surgery depended upon the decision of the multidisciplinary Committee of the Epilepsy Unit. The location of the presurgical EZ was determined by consensus during patient management meetings. Patients underwent comprehensive presurgical assessment which consisted of evaluation of a detailed clinical history and neurological examination, complete neuropsychological evaluation, psychiatric assessment, interictal, and ictal onset patterns in long-term scalp video-electroencephalogram (video-EEG), MRI, subtraction ictal-interictal single-photon emission computed tomography (SPECT) coregistered with MRI (SISCOM) and PET results. Invasive intracranial monitoring with subdural electrodes was performed when the standard presurgical evaluation failed to localize the EZ or when functional mapping of eloquent areas was needed. Histopathological findings and at least 1-year of follow-up after surgery according to Engel's classification scale were recorded. For inclusion in the study, a good to excellent post-operative outcome (Englel scale I-II) was required.

This validation study included 30 interictal ^18^F-FDG PET studies performed as part of the clinical work-up. The mean age of the patients was 36.0 years [standard deviation (SD) 11.5 years]; 66.7% (20/30) were female and 33.3% (10/30) male. The mean duration of epilepsy was 22.0 years (SD 13.1 years). In 25 patients, the presurgical EZ was determined by clinical and neuropsychologic data, video-EEG and neuroimaging, while invasive monitoring was needed in 5 patients. The presurgical EZ was located in the temporal lobe in 90.0% (27/30) of patients, 56.6% (17/30) of whom had medial TLE, 16.7% (5/30) neocortical TLE, and 16.7% (5/30) had both mesial and lateral TLE. In the remaining 3 patients, the presurgical EZ was located in the frontal, the parietal and the occipital lobe, respectively. PET studies were performed in all patients whose MRI was: (1) normal (*n* = 3); (2) unspecific or equivocal (*n* = 11), usually doubtful dysplasias or signal alterations; or (3) lesional (*n* = 16). In the latter case, PET imaging was performed when lesions were discordant with video-EEG (*n* = 1) or for delineation of the area to be resected (*n* = 15). Of these 16 patients with lesional MRI, 75.0% (12/16) had mesial temporal sclerosis, 12.5% (2/16) had areas of gliosis, 6.3% (1/16) had focal cortical dysplasia, and 6.2% (1/16) had dysembryoplastic neuroepithelial tumor. In the pathological study, mesial temporal sclerosis was found in 46.7% (14/30) of patients, focal cortical dysplasia in 13.3% (4/30), both mesial temporal sclerosis and focal cortical dysplasia in 13.3% (4/30), gliosis in 23.3% (7/30), and pleomorphic xanthoastrocytoma in 3.4% (1/30) of patients. The MRI of this latter patient was the study interpreted as dysembryoplastic neuroepithelial tumor. The post-operative seizure outcome was excellent (Engel I) in 83.3% (25/30) of patients and good (Engel II) in 16.7% (5/30). The mean duration of post-surgical follow-up was 3.6 years (SD 1.7 years).

The control group used in SPM and PET-analysis was the same that was used in our previous published studies (9).

This study was carried out in accordance with the Declaration of Helsinki. The protocol was approved by the hospital Ethics Committee and the need for written informed consent was waived. STARD guidelines were followed for reporting (10).

### Protocol for Imaging Acquisition and Reconstruction

The PET studies in both the patient and control groups were acquired and reconstructed following a routine clinical epilepsy protocol described previously (9, 11–13). Images were acquired in 3D mode with PET/CT equipment (Biograph; Siemens, Erlangen, Germany). Patients were required to rest quietly in a dimly lit room during the 40 min following ^18^F-FDG intravenous administration of approximately 5 MBq/kg. Subsequently, images were acquired using a standard 11-min schedule (1 min for CT and 10 min for PET). The ordered subset expectation maximization algorithm (16 subsets and 6 iterations) was used for PET data reconstruction with a matrix of 128 × 128 × 64 and a voxel size of 2.6 × 2.6 × 2.4 mm^3^. MRI studies were acquired with a 3-Tesla unit (Magnetom Trio; Siemens, Erlangen, Germany) using a specific epilepsy protocol.

Hypometabolic areas seen on PET studies appear as highlighted clusters after the statistical parametric analysis. A cluster is defined as a group of voxels (predetermined size—k) with a value lower than a predetermined statistical threshold. The statistical threshold (*p*-value) specifies the level of variation of activity considered significant to perform the image segmentation.

### SPM Processing

The PET studies were analyzed with the SPM8 software (Wellcome Department of Imaging Neuroscience, Institute of Neurology, London) (8). Reconstructed PET images were preprocessed by performing spatial normalization, proportional scaling intensity normalization and smoothing with a Gaussian kernel of full width at half maximum 8 × 8 × 8 mm^3^. Then, a two-sample *T*-Student test was carried out between the preprocessed PET image of each patient and the control database. To reduce background activity, only voxels with a value >30% of the maximum value (threshold mask of 0.3) were used in the statistical analysis. Statistical parametric maps were thresholded at *p* < 0.05 and cluster size k = 50, when family-wise error correction for multiple comparisons was performed, and at *p* < 0.001 and k = 100 otherwise. These threshold combinations were chosen because of their extensive use in previous studies (6, 14–16, 21, 22).

### PET-Analysis Processing

**Figure 1:**
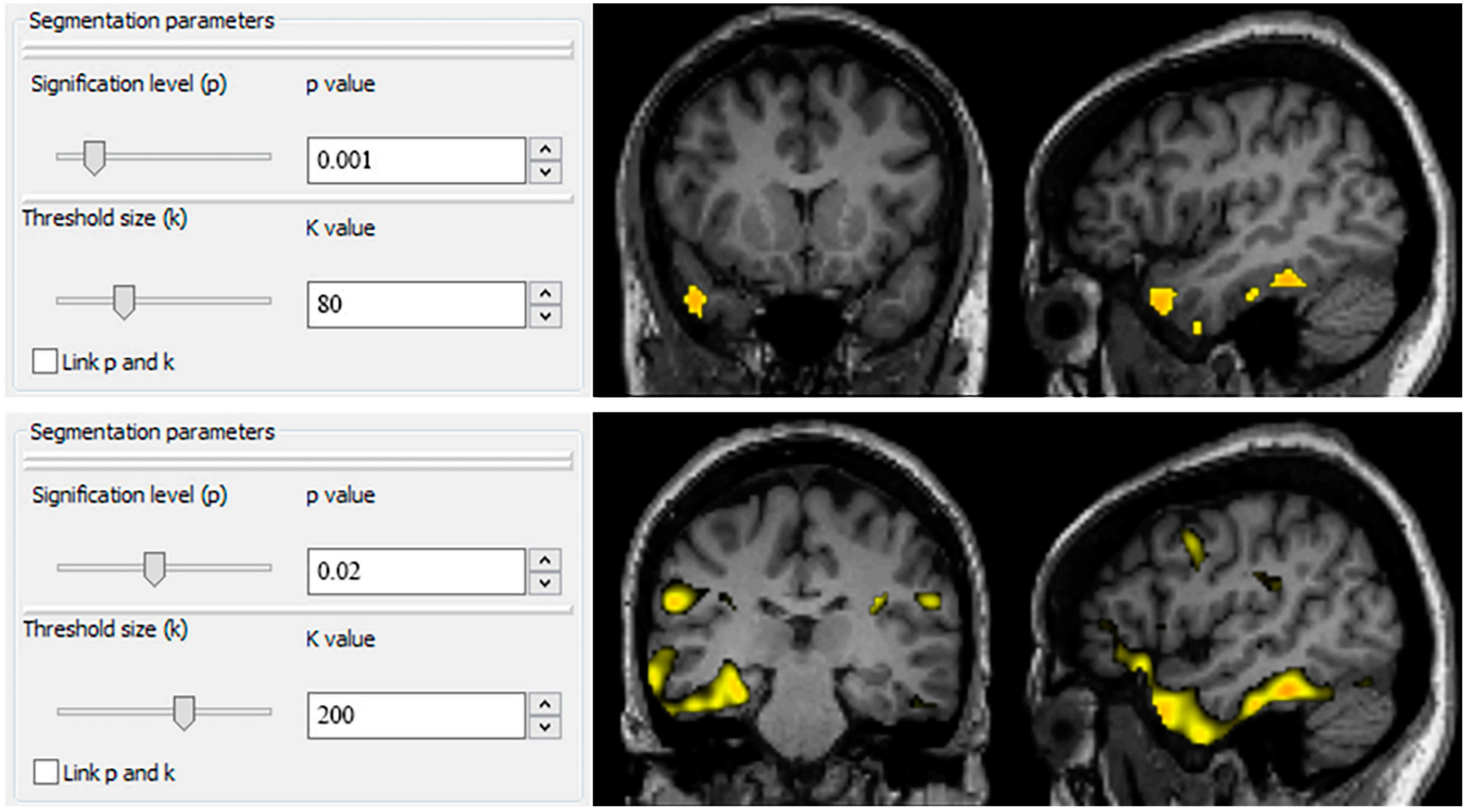
User interface of PET-analysis. Segmentation parameters can be rapidly switched simply by sliding any of the two scrollbars or using the up/down arrowheads representing p and k values. PET-analysis facilitates a more dynamic segmentation of images by allowing observers to modulate thresholds in real time, being more restrictive or liberal depending on the results of previous chosen thresholds.

PET-analysis is a new software based on the same methodology as the one carried out when SPM is used to perform a voxel-by-voxel statistical analysis. We introduced some methodological improvements which we thought were necessary for PET studies in the field of epilepsy based on our daily clinical experience. First, the PET studies were spatially normalized into a standard space with the aid of a previously created ^18^F-FDG PET template image (9) using a 12-parameter affine transformation followed by a non-linear deformation as in SPM normalization. Spatial normalization included linear and non-linear deformations and it was carried out with Elastix (14). Second, spatially normalized PET studies were intensity normalized in order to remove global intensity differences in cerebral metabolism between subjects (15). Intensity normalization was performed following a method that fits a parabola around the maximum value of the quotient distribution between images to overcome the bias in the normalization factor that can occur when the factor is calculated as the quotient between the total counts in the patient and the control studies. Third, the PET studies obtained in the previous step were smoothed with a Gaussian filter (full width at half maximum = 8 × 8 × 8 mm^3^) in order to reduce noise. Finally, a two sample *T*-Student test was performed in which one group consisted of the preprocessed PET study and the other group included the control studies, both groups after following the three steps described above. As seizures occur in the gray matter, a mask on which non-zero values were located only in the gray matter regions of the image was used in this comparison. Then, the parametric T-maps were thresholded with multiple *p*-values (ranging from 0.05 to 0.0001) and minimum clusters size (ranging from 50 to 200 k) combinations by sliding two scrollbars, as this new application rapidly processes different threshold combinations in the same workflow session (Figure 1). Segmentation parameters can be rapidly switched simply by sliding any of the two scrollbars or using the up/down arrowheads representing *p* and k values. PET-analysis facilitates a more dynamic segmentation of images by allowing observers to modulate thresholds in real time, being more restrictive or liberal depending on the results of previous chosen thresholds.

### Image Interpretation

Two experienced nuclear medicine physicians, who had no knowledge of the patients' clinical data, independently evaluated the PET studies and provided a presurgical EZ (hemispheric and sublobar location) for each set of PET imaging modalities (visual interpretation, SPM, and PET-analysis) obtained per patient. Processed PET images where corregistered with MRI following the same transformation and an automatic anatomic labeling template was used to determine the location of hypometabolic areas, represented as clusters (16). PET studies in which a hypometabolic area within the cerebral cortex was observed by visual interpretation or a cluster after SPM and PET-analysis processing were labeled as “positive studies.” When a unique cluster appeared after the analysis with SPM and PET-analysis, this area was defined as the presurgical EZ. When more than one cluster appeared after image processing, the definition of the presurgical EZ was based on both the statistical significance and the cluster size; the biggest cluster and with the highest statistical significance was considered as the presurgical EZ. Areas of decreased metabolism located outside the cerebral cortex or at the interhemispheric region were excluded. Any discrepancy in observer assessment was resolved by consensus. The presurgical EZ with side and sublobar concordance with the post-surgically confirmed EZ were considered as “correctly localizing studies.” The observers also recorded for each patient evaluation the number of clusters which were present in SPM and PET-analysis and the level of confidence with which the presurgical EZ for each study (high, medium or low confidence) was assigned.

MRI studies were visually interpreted by a senior neuroradiologist specialized in epilepsy.

### Analysis and Statistics

The proportion of positive and correctly localizing studies by visual assessment and after SPM and PET-analysis processing was compared with the McNemar's test. The proportion of positive and correctly localizing studies by MRI were also calculated. The concordance between the presurgical EZ according to the different techniques and the post-surgical EZ was evaluated using the kappa index (κ) and its 95% confidence interval (CI95%). This concordance was considered as slight with κ > 0 and ≤0.20, fair with κ > 0.21 and κ ≤ 0.40, moderate with κ > 0.41 and κ ≤ 0.60, substantial with κ > 0.61 and κ ≤ 0.80, and excellent with κ > 0.81. The mean value of the clusters obtained with SPM and PET-analysis was assessed. The proportion of correctly localizing PET studies was compared with the number of clusters and the level of confidence using the *Chi*^2^ test. The number of clusters and the level of confidence were compared with the analysis of variance (ANOVA). Differences were considered to be significant with a *p* < 0.05. Statistical analysis was performed with MedCalc Statistical Software version 16.2.1.

## Results

### Positive and Localizing Studies

Table 1 shows PET and MRI findings, histopathology and surgical outcome of each of the 30 patients included. Table 2 shows the number and percentage of positive, negative, correctly localizing, and non-localizing PET studies obtained after visual assessment, SPM, PET-analysis, and MRI. Hypometabolic areas were detected by visual assessment in 28/30 (93.3%) of PET studies, and the presurgical EZ was correctly localized in 21/30 (70.0%) of patients. PET-analysis achieved comparable results with 30/30 (100.0%) of positive and 20/30 (66.7%) correctly localizing studies. The SPM threshold *p* < 0.001/k = 100 obtained inferior results with 18/30 (60.0%) of positive and 11/30 (36.7%) correctly localizing studies. Statistically significant differences were found between this SPM threshold and the results achieved by visual assessment (*p* = 0.0309) and PET-analysis (*p* = 0.0117). The SPM threshold *p* < 0.05/k = 50 with family-wise error correction was too restrictive and only one study was positive, albeit non-localizing, after analysis (Table 1, patient 15). The 16/30 (53.3%) MRI were correctly localizing studies.

**Table 1:** PET and MRI findings, histopathology and surgical outcome.

**No**.	**PEZ**	**Visual**	**SPM1**	**SPM2**	**PET-a**	**MRI**	**Hp**.	**Engel**
1	R/MT	R/LT	Neg	R/LT	R/MT	R/MTS	MTS	I
2	L/MT	L/MT	Neg	L/LT	L/MT	L/MTS	MTS	I
3	L/MT	L/MT	Neg	L/MT	L/MT	L/MTS	MTS	I
4	L/MT	L/MT	Neg	L/LT	L/MT	L/MTS	MTS	I
5	L/MLT	L/LT	Neg	Neg	L/MT	L/MTS	FCDI+MTS	I
6	L/MT	L/MT	Neg	L/MT	L/MT	L/MTS	FCDIIA	I
7	R/MT	R/MT	Neg	Neg	R/MT	R/MTS	MTS	I
8	R/MT	R/LT	Neg	R/MT	R/MT	R/MTS	MTS	I
9	L/MT	L/MT	Neg	Neg	L/MT	L/MTS	MTS	I
10	R/MT	R/MT	Neg	R/MT	R/MT	R/MTS	MTS	II
11	L/MT	L/MT	Neg	Neg	L/LT	L/MTS	Gliosis	I
12	R/MT	R/MT	Neg	R/MT	R/MT	R/MTS	MTS	I
13	L/LT	L/LT	Neg	Neg	R/I	L/LT DNET	Xanthoastr.	II
14	L/LT	L/LT	Neg	L/LT	L/LT	L/LT Gliosis	Gliosis	I
15	R/P	R/P	R/O	R/O	R/P	R/P FCD	FCDII	I
16	L/O	L/O	Neg	Neg	L/O	L/O Gliosis	Gliosis	I
17	R/MT	R/LT	Neg	R/LT	R/LT	Non-L	MTS	I
18	R/MLT	R/MT	Neg	Neg	R/MT	Non-L	FCDIIA+MTS	I
19	L/MLT	R/MT	Neg	Neg	L/MT	Non-L	FCDIIA+MTS	I
20	R/MT	R/LT	Neg	R/LT	L/LT	Non-L	MTS	II
21	R/MT	R/MT	Neg	Neg	L/MT	Non-L	Gliosis	I
22	R/MT	R/LT	Neg	R/MT	R/MT	Non-L	MTS	I
23	R/MT	R/MT	Neg	Neg	R/MT	Non-L	MTS	II
24	L/F	L/F	Neg	L/F	L/F	Non-L	FCDIIA	I
25	R/LT	R/LT	Neg	Neg	L/F	Non-L	FCDI	I
26	R/MLT	R/LT	Neg	R/LT	L/LT	Non-L	Gliosis	I
27	L/MT	L/LT	Neg	L/MT	L/MT	Non-L	MTS	I
28	R/MLT	R/MT	Neg	Neg	L/LT	Non-L	FCDIIA+MTS	II
29	R/LT	Neg	Neg	L/P	L/LT	Non-L	Gliosis	I
30	R/LT	Neg	Neg	R/LT	R/LT	Non-L	Gliosis	I

*No., Patient number; PEZ, Post-surgically confirmed epileptogenic zone; Visual, PET visual assessment; SPM1, corrected p < 0.05 and k = 50; SPM2, uncorrected p < 0.001 and k = 100; PET-a, PET-analysis; Hp., Histopathology; F, Female; M, Male; R, Right; L, Left; MT, Mesial temporal; LT, Temporal; MLT, Mesial and lateral temporal; P, Parietal; O, Occipital; F, Frontal; I, Insula; Neg, Negative; Non-L, Non-lesional MRI (unspecific and negative studies); MTS, Mesial temporal sclerosis; DNET, dysembryoplastic neuroepithelial tumor; FCD, Focal cortical dysplasia; Xanthoastr., Pleomorphic xanthoastrocytoma*.

**Table 2:** Number and percentage of positive, negative, correctly localizing, and non-localizing PET studies by visual assessment, SPM and PET-analysis and MRI.

	**Positive (%)**	**Negative (%)**	**Localizing (%)**	**Non-localizing (%)**
Visual	28/30 (93.3)	2/30 (6.7)	21/30 (70.0)	7/30 (23.3)
SPM1	1/30 (3.3)	29/30 (96.7)	0/30 (0.0)	30/30 (100.0)
SPM2	18/30 (60.0)	12/30 (40.0)	11/30 (36.7)	7/30 (23.3)
PET-analysis	30/30 (100.0)	0/30 (0.0)	20/30 (66.7)	10/30 (33.3)
MRI	16/30 (53.3)	14/30 (46.7)^*^	16/30 (53.3)	0/30 (0.0)

* *Non-lesional MRI studies are shown in this square (unspecific and negative studies). Visual, PET visual assessment; SPM1, corrected p < 0.05 and k = 50; SPM2, uncorrected p < 0.001 and k = 100*.

Four patients (13.3%) with negative or incorrectly localizing PET by visual assessment and non-lesional by MRI had correctly localizing PET studies with post-processing techniques (Table 1, patients 19, 22, 27, and 30), one of them only with PET-analysis (patient 19). On the other hand, two patients (6.7%) who had correctly localizing PET by visual interpretation and MRI (Table 1, patients 11 and 13) were negative in SPM-analysis and had incorrectly localizing studies with PET-analysis. Among three patients (10.0%) in whom no SPM threshold combination, PET-analysis, or MRI showed any remarkable finding (Table 1, patients 17, 20, and 29), two were positive, although non-localizing, by visual assessment (patients 17 and 20) and the other patient also had a visually negative PET study (patient 29).

### Concordance With Post-surgical EZ

Table 3 shows the concordance between visual assessment, SPM and PET-analysis in comparison to the post-surgical EZ. The concordance was substantial for PET-analysis (κ = 0.643, CI 95% 0.439, 0.847) and visual interpretation (κ = 0.622, CI 95% 0.367, 0.877), while being fair for the SPM threshold *p* < 0.001/k = 100 (κ = 0.242, CI 95% 0.028, 0,455) and moderate for MRI (κ = 0.520, CI 95% 0.268, 0.772).

**Table 3:** Concordance between visual assessment, SPM, and PET-analysis compared to the post-surgical EZ.

	**Kappa index**	**CI_**95%**_**
Visual	0.622	0.367,0.877
SPM^a^	0.242	0.028,0.455
PET-analysis	0.643	0.439,0.847
MRI	0.520	0.268,0.772

* *SPM thresholded at uncorrected p < 0.001 and k = 100*.

*Visual, PET visual assessment*.

### Number of Clusters and Level of Confidence

The only study which was positive with the SPM threshold *p* < 0.05/k = 50 had one cluster, and the observers assigned the presurgical EZ with a high level of confidence. A mean of 2.6 clusters (SD 2.4 clusters) was observed with the SPM threshold *p* < 0.001/k = 100. The level of observer confidence for assigning the presurgical EZ with this SPM threshold combination was high in 10/30 (33.3%) studies, medium in 6/30 (20%), and low in 2/30 (6.7%) studies. Statistically significant differences were found between the proportion of correctly localizing studies vs. the number of clusters found in the analysis (*p* = 0.041) and the observer level of confidence (*p* = 0.024) with this SPM threshold *p* < 0.001/k = 100.

A mean of 3.2 clusters was observed with PET-analysis (SD 1.3 clusters). The level of confidence with which the observers assigned the presurgical EZ with PET-analysis was high in 13/30 (43.3%) studies, medium in 13/30 (43.3%), and low in 4/30 (13.4%) studies. There were statistically significant differences between the number of clusters and the proportion of correctly localizing studies (*p* = 0.043) and observer level of confidence (*p* = 0.038) in PET-analysis.

## Discussion

Assessment of medically refractory epilepsy is challenging and objective tools are needed to complement the interpretation of neuroimaging studies on visual assessment. The results of the present study have validated PET-analysis as a new software to objectively localize the post-surgically confirmed EZ, in a series of 30 operated patients who underwent an ^18^F-FDG PET study in the presurgical evaluation of drug-resistant epilepsy. PET-analysis was correctly localizing in two thirds of studies with substantial concordance with the post-surgical EZ, being comparable to the results achieved by visual assessment and significantly higher than the rate of correctly localizing results obtained with SPM, which showed fair concordance with the post-surgical EZ.

The concept of this new software PET-analysis arose from the need for an application which could rapidly process different threshold combinations in the same workflow session, simply by sliding two scrollbars representing a wide spectrum of values in terms of statistical significance and cluster sizes. PET-analysis facilitates a more dynamic segmentation of images by allowing observers to modulate uncorrected thresholds in real time, being more restrictive or liberal depending on the results of previous chosen thresholds. Moreover, PET-analysis is a SPM-independent application based on open-source software programs with a more intuitive graphical user interface for physicians not familiar with programming language.

The overall utility of SPM in PET studies of epilepsy is controversial; while some studies have shown that SPM has a tendency to improve visual assessment (17–20), other articles have reported similar (21–23) or even worse results (4, 24). In our study, visual assessment was superior to SPM with 70.0% (21/30) vs. 36.7% (11/30) of correctly localizing studies, respectively. However, these results may be attributable to the fact that only two thresholds were used in SPM, one of which was too restrictive, and the performance of SPM is dependent on the thresholds chosen for its analysis. Earlier publications in the literature, in both pediatric and adult patients, used fixed sizes of contiguous voxels for different *p*-values (6, 18–20) or exclusively one threshold combination (4, 21–23), which might not be suitable for all imaging studies. In the present study in which a new software was validated, we decided to apply the most common SPM thresholds used in the literature, which are a corrected *p*-value of *p* < 0.05, an uncorrected *p*-value of *p* < 0.001 and cluster sizes of 50–100 voxels, respectively (6, 17–20, 24, 25). However, as reported in our previous article (9), the greatest sensitivity and specificity for larger spatial extent of voxels tends to be achieved more significant *p*-values because multiple clusters might appear with less significant *p*-values. Inversely, for stricter *p*-values, smaller cluster sizes should be chosen since otherwise the hypometabolic regions would not likely survive the statistical analysis. According to our previous study, the best thresholds for SPM were an uncorrected *p*-value of *p* < 0.001 with k = 100 and an uncorrected *p*-value of *p* < 0.005 with k = 200. It would seem that the best approach in epilepsy would be to use uncorrected thresholds as the lesions in this disorder are usually small-sized areas of decreased metabolism which do not survive the statistical analysis when using corrected thresholds. Another remarkable point that should be mentioned is that some previous studies only included pediatric patients (19, 24, 25) and other articles are more heterogeneous and included mostly adult patients but also few children (6, 17, 18, 20). Although these articles used similar or even the same thresholds for SPM analysis, as ^18^F-FDG brain metabolism in children may differ from adults, it should be analyzed in further studies if different thresholds should be used depending on the age of the patient.

**Figure 2:**
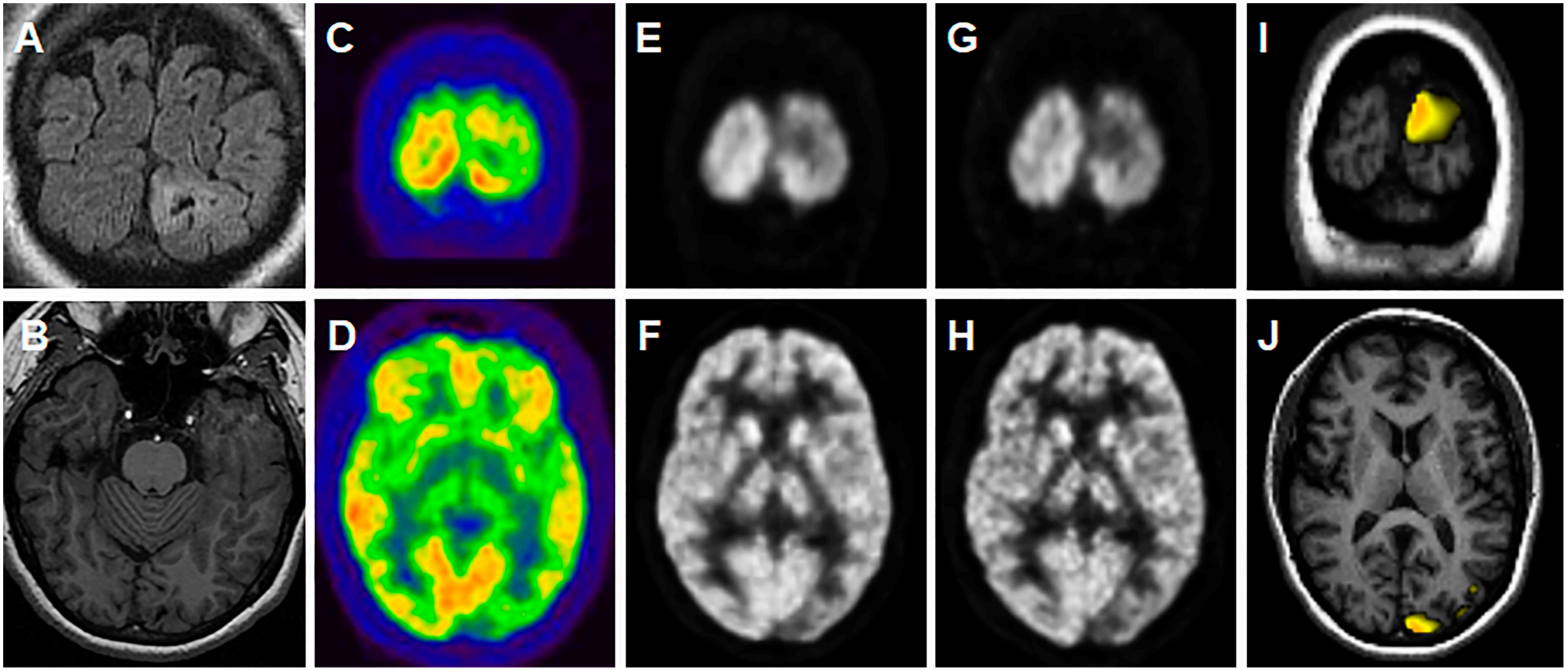
A representative case of the utility of PET-analysis. Patient with a 34-year history of medically refractory epilepsy with weekly seizures (Table 2, patient 16). Video-EEG showed epileptiform activity in the left occipital region. MRI depicted a focal lesion in the left occipital lobe **(A,B)** which was suggestive of a small area of residual encephalomalacia or secondly, cortical dysplasia with subcortical extension (MRI sequences from up to down: coronal FLAIR and axial contrast-enhanced T1-weighted). On suspicion of dysplasia, a PET study was requested to plan the extent of surgical resection, and a left occipital hypometabolism was seen on visual assessment **(C,D)**. No hypometabolic areas were present at any threshold in SPM analysis, although a left occipital hypometabolism was seen in the SPM-normalized images **(E,F)**, which was more evident in the PET-analysis-normalized images **(G,H)**. A left occipital hypometabolic area appeared on PET-analysis (thresholds shown: *p* = 0.008 and k = 200), corresponding with the occipital lesion on corregistered images with T1-weighted MRI **(I,J)**. The lesion was surgically resected and the pathology study diagnosed a chronic hemorrhagic necrosis foci with gliosis. Fifty-nine months years after surgery the patient is completely seizure-free (Engel I).

The performance of PET-analysis in this validation study was superior to that of SPM (Figure 2), with 66.7% (20/30) vs. 36.7% (11/30) of correctly localizing studies and substantial to fair concordance with post-surgical EZ, respectively. These better results could be ascribed to optimized thresholding in real time and spatial normalization. On the other hand, PET-analysis results were similar to visual assessment, although PET-analysis obtained a slightly superior concordance with the post-surgical EZ (κ = 0.643 vs. κ = 0.622, respectively). In accordance with the results of Zhu et al. (25), we believe that objective post-processing applications are complementary to visual assessment. These authors reported that visual assessment combined with SPM analysis detected more patients with abnormal glucose metabolism in PET studies compared to visual assessment alone. In our study, four patients (13.3%) who had negative or incorrectly localizing PET by visual assessment and non-lesional MRI (Table 1, patients 19, 22, 27, and 30) had correctly localizing PET studies with post-processing techniques, one of which (patient 19) was only obtained with PET-analysis. However, on the other hand, two patients (6.7%) who had correctly localizing PET by visual interpretation and MRI (Table 1, patients 9, 11, and 13) had incorrectly localizing studies with PET-analysis.

It is also noteworthy that despite SPM interpretation being considered user-independent, various significant hypometabolic areas may appear after analysis, especially when liberal thresholds are used, which may diminish observer confidence to determine the presurgical EZ. PET-analysis thresholds can be dynamically modulated in real time so that observers can apply more restrictive thresholds in order to decrease the number of clusters and, thus, increase their confidence when assigning the presurgical EZ among other hypometabolic areas. In other words, by decreasing the number of clusters the error range also reduces and, hence, the reliability of the test increases. Indeed, we found that the numbers of clusters in the PET-analysis correlated with the level of confidence and the rate of correctly localizing studies. Although the mean number of clusters obtained with PET-analysis was slightly higher than that of SPM (3.2 vs. 2.6 cluster per study), there was an increase in observer confidence with PET-analysis, with a high and medium level of confidence in 13/30 (43.3%) and 13/30 (43.3%) studies, respectively, vs. 10/30 (33.3%) and 6/30 (20%) with SPM. This higher level of confidence with PET-analysis could be explained by observers being able to modulate segmentation parameters depending on the hypometabolic areas seen with previously used thresholds, and thereafter choosing more restrictive or liberal *p*-values and cluster sizes. According to the results of this study and also to our clinical experience, the best approach in epilepsy is to use multiple uncorrected thresholds as some lesions are small-sized areas of decreased metabolism which are eliminated when using corrected thresholds. SPM is a generic program designed for the analysis of brain imaging data and PET-analysis was created to specifically process PET images from epileptic patients. It is also important to mention that the statistical test, which is a *T*-Student test comparison, is only performed once per patient and thus the use of a correction for multiple tests is not applicable in this situation.

This study had some limitations, the first being a selection bias inherent to the retrospective study design in a very selected group of patients. We needed cases with a confirmed lesion culprit of epilepsy and for this reason we could only include operated patients who were seizure free during a long follow-up, as only under these circumstances the EZ can be defined. Another limitation implicit in a parametric analysis is that it is very possible that ^18^F-FDG brain uptake of an individual patient may differ from the control group in a number of brain areas, related to the EZ or not. To validate this new application, most of the PET studies included had evident presurgical EZ on visual assessment to confirm that PET-analysis was able to detect these unambiguous hypometabolisms. It remains to be determined if PET-analysis is capable of prospectively detecting more subtle hypometabolisms that could be hidden to the naked eye in a non-surgically intervened group of patients, who are more representative of the clinical heterogeneity found in routine practice. Objective tools like PET-analysis could help the neurologist and the neurosurgeon in identifying the EZ but have to be considered together with other imaging modalities and additional patient data such as the semiology of the epileptic seizures and video-EEG, all of which should be performed as part of the regular preoperative work-up and evaluated in a multidisciplinary committee.

In conclusion, PET-analysis is a robust in-house application for EZ localization in PET studies of patients with medically refractory epilepsy. This new software showed a better performance than SPM with the fixed standard parameters, as it can easily and rapidly process different thresholds of image segmentation which can be switched in real time. It also has an intuitive graphical user interface making it user-friendly for physicians. Although this software has to be tested in a bigger and non-selected group of patients, the results of the present proof-of-concept study validate the use of PET-analysis as an objective complementary tool to visual assessment for EZ localization.

## Ethics Statement

This study was carried out in accordance with the Declaration of Helsinki. The protocol was approved by the Ethics Committee of the Hospital Clinic of Barcelona and the need for written informed consent was waived. STARD guidelines were followed for reporting.

## Author Contributions

MM, AN-B, JP, and XS contributed in the conception and design of the study. MM and AN-B participated as well in acquiring and processing data. MM performed the statistical analysis. MM drafted the manuscript and AN-B and XS were major contributors in writing the manuscript. MM, AN-B, AP, JP, DR, FP, and XS critically revised the manuscript and enhanced its intellectual content. All authors made a substantial contribution to analyzing and interpreting data and revised and approved the final manuscript.

### Conflict of Interest Statement

The authors declare that the research was conducted in the absence of any commercial or financial relationships that could be construed as a potential conflict of interest.

## Abbreviations

CI95%: 95% confidence interval
EZ: epileptogenic zone
SPM: statistical parametric mapping
TLE: temporal lobe epilepsy.

## References

[1] Feng R Hu J Pan L Shi J Qiu C Lang L. Surgical treatment of MRI-negative temporal lobe epilepsy based on PET: a retrospective cohort study. Stereotact Funct Neurosurg. (2014) 92:354–9. 10.1159/000365575 25358872

[2] Gok B Jallo G Hayeri R Wahl R Aygun N. The evaluation of FDG-PET imaging for epileptogenic focus localization in patients with MRI positive and MRI negative temporal lobe epilepsy. Neuroradiology. (2013) 55:541–50. 10.1007/s00234-012-1121-x 23223825

[3] LoPinto-Khoury C Sperling MR Skidmore C Nei M Evans J Sharan A. Surgical outcome in PET-positive, MRI-negative patients with temporal lobe epilepsy. Epilepsia. (2012) 53:342–8. 10.1111/j.1528-1167.2011.03359.x 22192050

[4] Chassoux F Rodrigo S Semah F Beuvon F. FDG-PET improves surgical outcome in negative MRI Taylor-type focal cortical dysplasias. Neurology. (2010) 75:2168–75. 10.1212/WNL.0b013e31820203a9 21172840

[5] Chandra PS Vaghania G Bal CS Tripathi M Kuruwale N Arora A. Role of concordance between ictal-subtracted SPECT and PET in predicting long-term outcomes after epilepsy surgery. Epilepsy Res. (2014) 108:1782–9. 10.1016/j.eplepsyres.2014.09.024 25308754

[6] van't Klooster MA Huiskamp G Zijlmans M Debets RMC Comans EFI Bouvard S. Can we increase the yield of FDG-PET in the preoperative work-up for epilepsy surgery? Epilepsy Res. (2014) 108:1095–105. 10.1016/j.eplepsyres.2014.04.011 24893829

[7] Brodbeck V Spinelli L Lascano AM Wissmeier M Vargas M-I Vulliemoz S. Electroencephalographic source imaging: a prospective study of 152 operated epileptic patients. Brain. (2011) 134:2887–97. 10.1093/brain/awr243 21975586 PMC3187544

[8] Acton PD Friston KJ. Statistical parametric mapping in functional neuroimaging: beyond PET and fMRI activation studies. Eur J Nucl Med. (1998) 25:663–7. 9741993

[9] Mayoral M Marti-Fuster B Carreño M Carrasco JL Bargalló N Donaire A Seizure-onset zone localization by statistical parametric mapping in visually normal ^18^F-FDG PET studies. Epilepsia. (2016) 57:1236–44. 10.1111/epi.13427 27286896

[10] Bossuyt PM Reitsma JB Bruns DE Gatsonis CA Glasziou PP Irwig LM. The STARD statement for reporting studies of diagnostic accuracy: explanation and elaboration. Clin Chem. (2003) 49:7–18. 10.1373/49.1.7 12507954

[11] Rubí S Setoain X Donaire A Bargalló N Sanmartí F Carreño M. Validation of FDG-PET/MRI coregistration in nonlesional refractory childhood epilepsy. Epilepsia. (2011) 52:2216–24. 10.1111/j.1528-1167.2011.03295.x 22050207

[12] Perissinotti A Setoain X Aparicio J Rubí S Fuster BM Donaire A Clinical role of subtraction ictal SPECT coregistered to MR imaging and ^18^F-FDG PET in pediatric epilepsy. J Nucl Med. (2014) 55:1099–105. 10.2967/jnumed.113.136432 24799620

[13] Fernández S Donaire A Serès E Setoain X Bargalló N Falcón C. PET/MRI and PET/MRI/SISCOM coregistration in the presurgical evaluation of refractory focal epilepsy. Epilepsy Res. (2015) 111:1–9. 10.1016/j.eplepsyres.2014.12.011 25769367

[14] Klein S Staring M Murphy K Viergever MA Pluim JPW. elastix: a toolbox for intensity-based medical image registration. IEEE Trans Med Imaging. (2010) 29:196–205. 10.1109/TMI.2009.2035616 19923044

[15] Martí Fuster B Esteban O Planes X Aguiar P Crespo C Falcon C. FocusDET, a new toolbox for SISCOM analysis. Evaluation of the registration accuracy using Monte Carlo simulation. Neuroinformatics. (2013) 11:77–89. 10.1007/s12021-012-9158-x 22903439 PMC3538012

[16] Tzourio-Mazoyer N Landeau B Papathanassiou D Crivello F Etard O Delcroix N. Automated anatomical labeling of activations in SPM using a macroscopic anatomical parcellation of the MNI MRI single-subject brain. Neuroimage. (2002) 15:273–89. 10.1006/nimg.2001.0978 11771995

[17] Kim YK Lee DS Lee SK Chung CK Chung J-K Lee MC ^18^F-FDG PET in localization of frontal lobe epilepsy: comparison of visual and SPM analysis. J Nucl Med. (2002) 43:1167–74. 12215554

[18] Plotkin M Amthauer H Merschhemke M Lüdemann L Hartkop E Ruf J Use of statistical parametric mapping of ^18^F-FDG-PET in frontal lobe epilepsy. Nuklearmedizin. (2003) 42:190–6. 10.1267/NUKL03050190 14571315

[19] Kumar A Juhász C Asano E Sood S Muzik O Chugani HT. Objective detection of epileptic foci by ^18^F-FDG PET in children undergoing epilepsy surgery. J Nucl Med. (2010) 51:1901–7. 10.2967/jnumed.110.075390 21078805 PMC3157889

[20] Kim MA Heo K Choo MK Cho JH Park SC Lee JD. Relationship between bilateral temporal hypometabolism and EEG findings for mesial temporal lobe epilepsy: analysis of ^18^F-FDG PET using SPM. Seizure. (2005) 15:56–63. 10.1016/j.seizure.2005.11.007 16386927

[21] Lee DS Lee JS Kang KW Jang MJ Lee SK Chung J Disparity of perfusion and glucose metabolism of epileptogenic zones in temporal lobe epilepsy demonstrated by SPM / SPAM analysis on ^15^O water PET, [^18^F] FDG-PET, and [^99m^ Tc]-HMPAO SPECT. Epilepsia. (2001) 42:1515–22. 10.1046/j.1528-1157.2001.21801.x 11879361

[22] Lee JJ Kang WJ Lee DS Lee JS Hwang H Kim KJ. Diagnostic performance of ^18^F-FDG PET and ictal ^99m^Tc-HMPAO SPET in pediatric temporal lobe epilepsy: quantitative analysis by statistical parametric mapping, statistical probabilistic anatomical map, and subtraction ictal SPET. Seizure. (2005) 14:213–20. 10.1016/j.seizure.2005.01.010 15797357

[23] Wong CH Bleasel A Wen L Eberl S Byth K Fulham M. The topography and significance of extratemporal hypometabolism in refractory mesial temporal lobe epilepsy examined by FDG-PET. Epilepsia. (2010) 51:1365–73. 10.1111/j.1528-1167.2010.02552.x 20384730

[24] Archambaud F Bouilleret V Hertz-Pannier L Chaumet-Riffaud P Rodrigo S Dulac O. Optimizing statistical parametric mapping analysis of ^18^F-FDG PET in children. EJNMMI Res. (2013) 3:2. 10.1186/2191-219X-3-2 23289862 PMC3558387

[25] Zhu Y Wu S Hou H Ji J Zhang K Chen Q Glucose metabolic profile by visual assessment combined with SPM analysis in pediatric patients with epilepsy. J Nucl Med. (2017) 58:1293–99. 10.2967/jnumed.116.187492 28104740

